# Efficiency of porcine somatic cell nuclear transfer – a retrospective study of factors related to embryo recipient and embryos transferred

**DOI:** 10.1242/bio.20135983

**Published:** 2013-10-02

**Authors:** Yongye Huang, Hongsheng Ouyang, Hao Yu, Liangxue Lai, Daxin Pang, Zhanjun Li

**Affiliations:** Jilin Provincial Key Laboratory of Animal Embryo Engineering, College of Animal Sciences, Jilin University, Changchun 130062, China

**Keywords:** Somatic cell nuclear transfer, Pregnancy, Climate, Ovulation, Abortion

## Abstract

The successful generation of pigs via somatic cell nuclear transfer depends on reducing risk factors in several aspects. To provide an overview of some influencing factors related to embryo transfer, the follow-up data related to cloned pig production collected in our laboratory was examined. (i) Spring showed a higher full-term pregnancy rate compared with winter (33.6% vs 18.6%, *P* = 0.006). Furthermore, a regression equation can be drawn between full-term pregnancy numbers and pregnancy numbers in different months (*y* = 0.692*x*−3.326). (ii) There were no significant differences detected in the number of transferred embryos between surrogate sows exhibiting full-term development compared to those that did not. (iii) Non-ovulating surrogate sows presented a higher percentage of full-term pregnancies compared with ovulating sows (32.0% vs 17.5%, *P* = 0.004; respectively). (iv) Abortion was most likely to take place between Day 27 to Day 34. (v) Based on Life Table Survival Analysis, delivery in normally fertilized and surrogate sows is expected to be completed before Day 117 or Day 125, respectively. Additionally, the length of pregnancy in surrogate sows was negatively correlated with the average litter size, which was not found for normally fertilized sows. In conclusion, performing embryo transfer in appropriate seasons, improving the quality of embryos transferred, optimizing the timing of embryo transfer, limiting the occurrence of abortion, combined with ameliorating the management of delivery, is expected to result in the harvest of a great number of surviving cloned piglets.

## Introduction

Due to sharing many similarities with human beings, the combination of somatic cell nuclear transfer (SCNT) techniques and genetic modification technology to generate transgenic pigs can be widely applied in biomedical and basic research. Therefore, many research groups are devoted to this work and much progress has been made during the past several years. Despite the fact that many genetically modified cloned pigs have been generated (Lai et al., 2002; Lai et al., 2006; Park et al., 2001; Rogers et al., 2008; Suzuki et al., 2012), there are still problems that need to be resolved and the efficiency of porcine SCNT is still quite low. The successful generation of cloned pigs via SCNT can be impacted by many factors (Polejaeva et al., 2000; Vajta et al., 2007). For example, there is a minimal requirement of at least four viable embryos to establish a pregnancy in pigs (Polejaeva et al., 2000; Polge et al., 1966). It has been 13 years since the birth of the first pig generated via SCNT (Polejaeva et al., 2000), however, reports on systemic analysis of influencing factors related to embryo transfer were rare.

The successful generation of pigs via somatic cell nuclear transfer depends on reducing risk factors in several aspects. Therefore, to provide insights to assist the field of porcine SCNT, a retrospective study on the data related to the production of cloned pigs obtained in our laboratory was performed. The retrospective analysis was organized according to the possible impacts on the process of SCNT cloned pigs production: before embryo transfer, the proper number of transferred embryos and the extent of estrus (ovulation or not) for surrogate sows should be considered (**First subgroup analysis**); after embryo transfer, taking good care of the surrogate sows to reduce abortion but deliver more piglets is an important task (**Second subgroup analysis**); in addition, it is supposed that, no matter before or after embryo transfer, the process could be affected by the climate factors (**Principal analysis**). To conduct the analysis, three conceptions were firstly defined. The number of pregnancies (PR) is defined as the number of surrogate sows detected to be pregnant via ultrasound examination. The number of full-term pregnancies (FTP) denotes the number of surrogate sows giving birth to piglets. The number of blocked pregnancy (BLO) was calculated from the PR number minus the FTP number.

## Materials and Methods

### Ethics statement

All animal experiments were conducted according to the guidelines on animal care and use established by the Animal Care and Welfare Committee of Jilin University, with an approval number 2008-013.

### Study subjects

Data related to the production of cloned pigs was collected from 2008 to the first half of 2012 in our laboratory. Porcine ovaries were provided by HuaZheng Agriculture Development Co. Ltd., and the usage permission was also obtained from the company. Oocyte maturation, micromanipulation fusion, activation and embryo culturing were performed in Jilin Provincial Key Laboratory of Animal Embryo Engineering. Embryo transfer and pig farming were carried out in Original Breeding Pig Farm of Jilin University and HuiChang Livestock Co. Ltd. All these three places are located at Changchun in China (latitude 43°05′–45°15′N and longitude 124°18′–127°02′E).

### Measures

#### (i) Data for embryo transfer

The protocols for the generation of SCNT cloned pigs were based on those previously described in detail by Lai and Prather (Lai and Prather, 2003). According to the different experimental designs fetal fibroblast cells were transfected with different gene-expressing vectors and several kinds of transgenic cell lines were used as donor cells for SCNT each year. The day of SCNT was taken as Day 0. Nearly all of the embryo transfer were performed on Day 1 (448/462 = 97.0%); some embryo transfer were conducted on Day 2 (14/462 = 3.0%). All of the reconstructed embryos with good morphology were transferred. Naturally cycling sows were selected as surrogate one day after the onset of estrus in accordance with the behavior and genital swelling level of the sow through visual inspection.

For each embryo transfer, an elaborate record was made, including date time, ear number for surrogate sow, ovulation or not, the number of transferred embryos and so on. Ovulation or not was recorded at the time of the surgical operation for embryo transfer being performed. After surgical operation, the surrogate sows were elaborate fed and observed every day. The pregnancy detection was conducted on Day 23–27 via ultrasound examination. The occurrence of abortion was confirmed through observation of the excretion of abortion sample, such as amniotic sac and fetus; and the date for the occurrence of abortion was recorded. When the sow reached a full-term development, the date for the delivery and litter size was also recorded. As a control, the records of normal fertilized sow deliveries in 2011 were examined.

#### (ii) Month and season designation

In the present study, each month and season corresponded to the date of initiation of the SCNT procedure that is the date of oocyte collection. To better determine the relationship between the generation of successfully cloned pigs and the climate, seasons were designated based on the Climate-Temperature Law (supplementary material Table S3) which was according to the daily average temperature. And the data for the average temperature was taken from the weather record of Changchun.

The winter here was as long as 5 to 6 months in each year analyzed basing on Climate-Temperature Law (supplementary material Table S4), which was longer that the pig's gestation period (about 114 days); therefore, to study the impact of climate on the final stage of gestation progress, the winter was firstly separated into two parts (1^st^ and 2^nd^ winter). The 1^st^ winter was defined as dates from the initial date of winter till December and the 2^nd^ winter from January to the end of winter. Actually, the embryo transfer was performed from March to November in our laboratory. In other words, the last stage of gestation also occurred in winter in the 1^st^ winter group, whereas it took place in late spring or in summer in the 2^nd^ winter group.

### Statistical analysis

All of the data were analyzed using SPSS version 16.0 software (SPSS Inc., Chicago, IL, USA), and when the *P* value less than 0.05 was taken as significant difference.

The PR rate and FTP rate between groups were determined using Chi-square Test, respectively. These groups included ovulating and non-ovulating group; spring, summer, autumn and winter group; 1^st^ and 2^nd^ winter group. In addition, the PR rate, FTP rate and BLO rate in each month was compared with the average percentage using Binomial Test, respectively. To better determine the most suitable months for conducting SCNT, Chi-square Test was also performed to compare the FTP rate in May and other months separately, and the one-tailed significance value was used for comparison.

The relationship was determined through Bivariate Correlations Test, between the number of embryos transferred and the litter size of surrogate sows; length of pregnancy and the number of born piglets per litter; and among the number of total surrogates, PR number and FTP number in different months. The relationship among the total surrogate numbers, FTP numbers and PR numbers was analyzed using Bivariate Correlations Test and Linear Regression Test based on months.

The mean number of transferred embryos between FTP and non-FTP group, and the average litter size between the surrogate sows and normal fertilized sows were compared using Independent-Samples T Test. The One-way ANOVA was applied when comparing the mean number among different stages of abortion and delivery, respectively.

To evaluate the complete rate of the abortion (opposite to survival proportion) and the delivery (equal to survival proportion) in each day point, Life Table Survival Analysis was applied, respectively.

## Results and Discussion

### Principal analysis: examining the influence of climate on the developmental process for cloned embryos

From 2008 to the first half of 2012, 462 embryo transfers were performed in our laboratory. Prior to analysis, Chi-square Test was conducted to examine the FTP rate among these five years (supplementary material Table S1). The results showed no significant differences from 2008 to the first half of 2012 in FTP rate (χ^2^ = 7.640, ν = 4, *P* = 0.106), indicating that there were no large fluctuations in these years and the following analysis could be reliable.

#### (i) Spring (10 to 22°C) is expected to be the best season for porcine embryo transfer

**Fig. 1.**
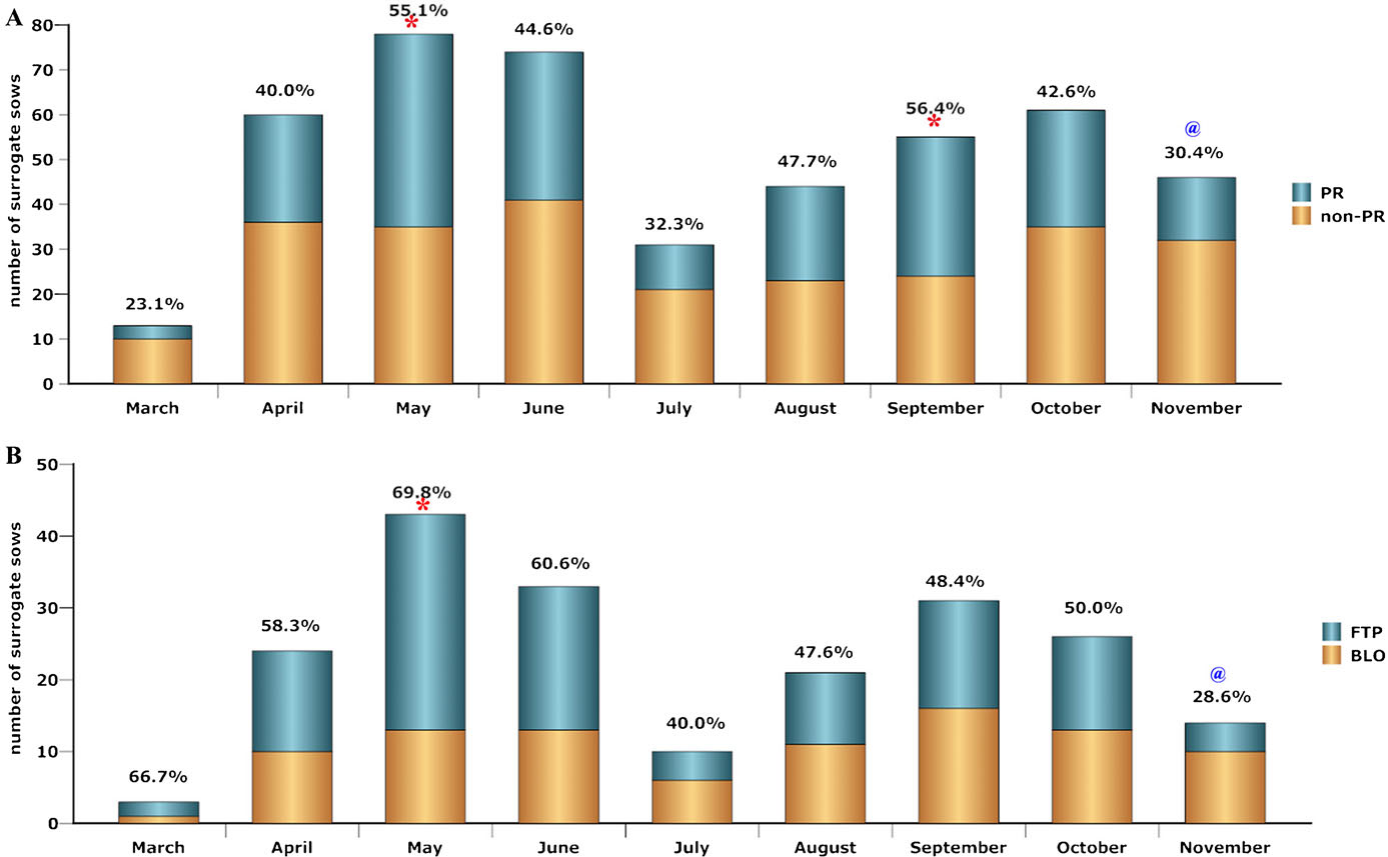
(A) The pregnancy (PR) number in the total surrogate number from March to November. The corresponding percentage for the PR number in the total surrogate number was indicated on the top of the bar. Comparison of PR rate in each month with the average level was made by Binomial tests (seen also in supplementary material Table S2). (B) The full-term development pregnancy (FTP) number in the PR number in different month. The corresponding percentage for the FTP number in the PR number was indicated on the top of the bar. Comparison of the ratio for the FTP number in the PR number in each month with the average level was made by Binomial tests (seen also in supplementary material Table S7); * denotes significantly higher than the average level, and ^@^ denotes significantly lower than the average level.

As shown in supplementary material Table S2, the PR rates between each month did not show significant differences, though it was high in May and September but low in November (Fig. 1A). However, the FTPs rates were significantly different in different months, being highest in May and lowest in November. Additionally, the FTP rates in April, July, October and November were significantly different from that in May (supplementary material Table S3). Thus, May, June, August and September are the most appropriate months for embryo transfer. Another implication of the above results appears to be that spring and autumn should be the favored seasons for embryo transfer. However, it should be noted that the climate at this time varies greatly among different areas of the world. Therefore, in the present study, seasons were designated based on the Climate-Temperature Law (supplementary material Table S4). As shown in Table 1, there were no significant differences detected in the PR rates among spring, summer and autumn; and the FTP rate in spring is higher than that in summer and winter. These results possibly suggested that spring and autumn are suitable seasons for SCNT. Another group has reported a similar result, showing that embryos transferred in spring (defined as March to May) were more likely to develop to term (Koo et al., 2010), which further confirmed that our analysis was reliable.

**Table 1.**
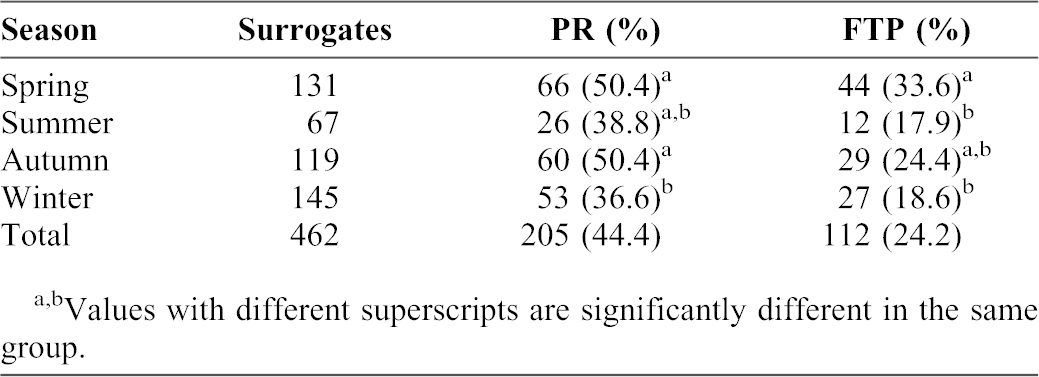
The statistics of pregnancy in different seasons.

It is well known that there is a high intracellular lipid content in porcine oocytes, which may make the oocyte sensitive to temperature (Li et al., 2006). In November and in winter, the loss of embryos during gestation was observed to be considerable, suggesting that cold weather is unfavorable for cloned pig production. Previous reports have shown that there are seasonal fluctuations in farrow rates (Hurtgen and Leman, 1980), with fertility during the summer–autumn period being reduced (Love et al., 1993). In addition, more blastocysts, as well as expanded blastocysts, were generated from oocytes collected in spring compared to those collected in winter in a porcine parthenogenesis experiment, although the rate of production of cloned piglets did not differ between the two seasons (Ma et al., 2009). However, summer in Changchun was short, and the temperature was relatively mild. Therefore, determination of the exact effect that a high temperature climate exerts on cloned pig production could be aided by including data from other laboratories.

#### (ii) Full-term development is not largely affected by the climate at the final stage of gestation

The above result about the effect of climate on the SCNT embryo development was revealed according to the date of initiation of the SCNT procedure. However, the question of whether the final stage of gestation progress is also susceptible to climate remains unanswered. Therefore, a statistical analysis was comparing the 1^st^ and 2^nd^ winter. The results suggested that no significant differences exist between the two groups, though the 2^nd^ winter presented a higher FTP percentage (supplementary material Table S5). These results may indicate that the climate is not as critical for the late stage of gestation. It is certainly also possible that the sample size is insufficiently large. Because domestic pigs are usually kept in house, we speculated that the most sensitive process to the climate should be the transportation of the ovary donor gilts to the slaughter house, in which gilts were exposed to the natural environment thus affecting their physiological conditions. In fact, the air composition and air circulation in the feeding house also varied in accordance with different seasons, because it is far from an independent system in which the temperature and illumination are strict regulated.

#### (iii) The occurrence rate for surrogate sow suffering a blocked pregnancy during gestation was practically stable

**Fig. 2.**
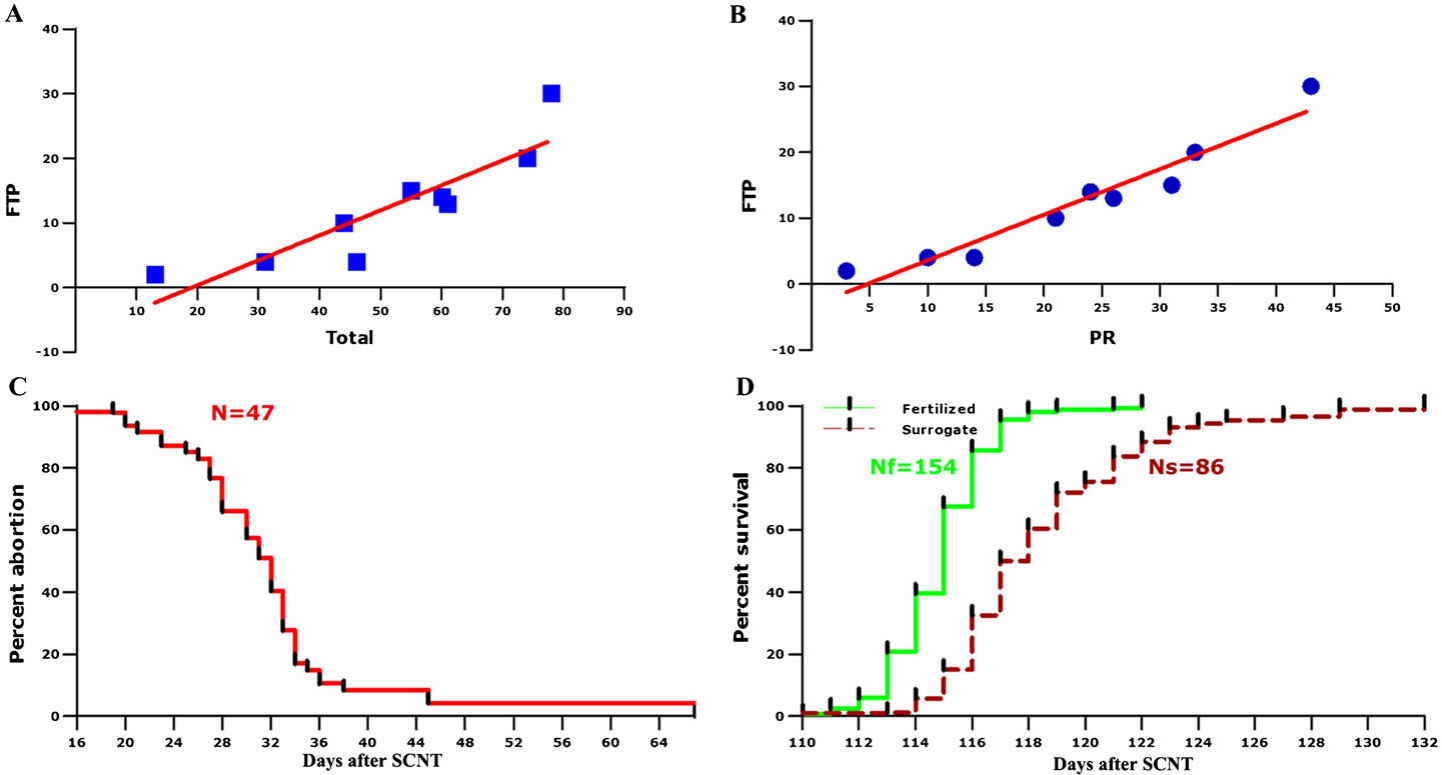
(A) Line drawing between total surrogate number and FTP number based on the statistics in different months. (B) Line drawing between PR number and FTP number based on the statistics in different months. (C) Distribution curve of abortions for 47 surrogate sows. N: the number of sows observed. (D) Survival curves comparing delivery between fertilized sows and surrogate sows. Nf: the number of fertilized sows; Ns: the number of surrogate sows.

It was shown that the relationship among the total surrogate numbers, FTP numbers and PR numbers in different months appeared to be positive (supplementary material Table S12). Therefore, linear regression tests were performed according to the numbers in different months (supplementary material Table S6). These tests showed a close relationship between FTP numbers and total surrogate numbers (*P* = 0.001). As revealed in Fig. 2A, the regression equation describing the relationship between FTP numbers (*y*) and total numbers (*x*) is *y* = 0.386*x*−7.387. This regression equation not only indicates that our system was stable but also suggests that more full-term pregnancies can be expected when the number of embryo transfer is increased. In addition, there was also a linear relationship observed between FTP numbers and PR numbers (Fig. 2B), for which the regression equation is as follows: *y* = 0.692*x*−3.326. In most cases, the PR numbers were much higher than the FTP numbers, and pregnancy is also expected to be blocked after implantation in many surrogate sows. Since embryo losses can hardly be avoided, the equation describing the relationship between FTP numbers and PR numbers will be useful for forecasting the outcomes of embryo transfer. It is also possible indicate that the occurrence rate of surrogate sows exhibiting a blocked pregnancy (BLO) was stable and independent of the PR rate. It should be noted that the BLO rate was significantly lower in May and higher in November when compared with the average level (Fig. 1B; supplementary material Table S7), suggesting that there was a small effect of climate fluctuations on fetal resorption and/or abortion (*P* = 0.226).

### First subgroup analyses: determining influencing factors before embryo transfer

#### (i) The number of transferred embryos might not be as important as other parameters

Due to the poor quality of the cloned embryos, people prefer to transfer a large amount of embryos into surrogate sows (Vajta et al., 2007). It seems that the surrogate sows with more transferred embryos would reach higher FTP rate. However, as shown in supplementary material Table S8, the number of transferred embryos in the FTP group did not show significant differences compared with the number in the non-FTP group. This finding may suggest that a wide range of the number of transferred embryos had the same opportunity to reach pregnancy. However, were the surrogate sows into which more embryos were transferred likely to give birth to more piglets? To answer this question, a correlation analysis was performed. The result indicated that there was no close correlation between the number of transferred embryos and the litter size (supplementary material Table S12). Actually, three live cloned piglets was harvested by transferring only 15 embryos at the 2–4 cell stage in one previous study (Hoshino et al., 2005). It is possible that the quality, rather than the quantity, of transferred embryos is much more important for the successful generation of SCNT pigs.

#### (ii) Surrogate sows to which SCNT embryos are transferred prior to ovulation exhibit a higher FTP percentage

As shown in Table 2, the PR rate was almost the same between these two groups, but the FTP percentage in the non-ovulating group was significantly higher than that in the ovulating group. Similar results were reported by the laboratory of Professor Byeong-Chun Lee (Koo et al., 2010). These results potentially suggest that the physiological environment in the oviduct and/or uterus of the ovulating sows was not highly suitable for embryo transferred at one-cell stage. Neuropeptides had been found to affect ovulation (Evans and Anderson, 2012), and plasma leptin concentrations are positively correlated with the ovulation rate on one day before and the day of estrus cycle (Gonzalez-Bulnes et al., 2012). Interestingly, leptin improves the porcine blastocyst-formation rate and total cell number of blastocysts derived from both parthenogenetic and SCNT embryos (Wei et al., 2009; Kun et al., 2007). Thus, it is possible that some compounds released prior to ovulation could improve the developmental competence of porcine embryos. However, these findings do not simply indicate that ovarian condition at the time of embryo transfer only affects the development of transferred embryos prior to the blastocyst stage. A previous study found that the percentage embryonic mortality among Day 6 embryos transferred into Day 7 recipients was higher than that of Day 7 embryos transferred into Day 6 recipients on Day 30 (Pope et al., 1986). However, it should be noted that the average litter size in both groups was almost the same and that the litter size in both groups ranged from 1 to 8 piglets.

**Table 2.**
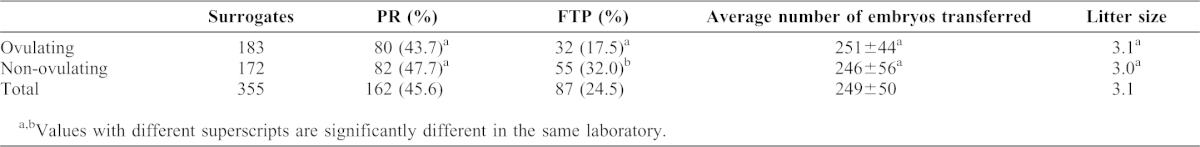
Effects of ovulating on pregnancy.

### Second subgroup analyses: management after embryo transfer

#### (i) Abortion was most likely to take place from Day 27 to Day 34

In the statistics, 47 surrogates were recorded to have experienced abortion (supplementary material Table S9). The abortions took place from Day 19 to Day 67 after SCNT. When a Life Table Survival Analysis was applied, the results showed that the median survival point corresponded to Day 32 (Fig. 2C), and the survival proportion was lower than 5% after Day 45. That is to say, abortion is expected to be accomplished before Day 45. Furthermore, it could be observed that the abortion was concentrated at Day 27 to Day 34 (supplementary material Table S9). Therefore, if designating this interval as the intermediate stage, the abortion day points could be separated into three stages, including early abortion (prior to Day 27) with a survival proportion higher than 80%, intermediate abortion (Day 27 to Day 34) with a survival proportion range from 80% to 20% and late abortion (after Day 34) with a survival proportion lower than 20%. When applying One-way ANOVA analysis, it further confirmed that the intermediate abortion stage (Day 27 to Day 34) represented the peak period of abortion (*P* = 0.000). Similarly, there was a previous study reporting that abortion and/or resorption took place between Day 25 and Day 40 of gestation, with mean Day 35±2, in hand-made cloned embryos (Schmidt et al., 2011).

#### (ii) Surrogate sows show a longer pregnancy period

Excluding the sows subjected to Caesarean delivery, 86 full-term surrogate sows and 154 fertilized sows were included in the statistics (supplementary material Table S10). The length of surrogate sow pregnancy ranged from 113 to 132 days, and 267 piglets were delivered, averaging approximately 3.10 piglets per litter. Among the fertilized sows, the length of pregnancy ranged from 110 to 122 days, with an average litter size of 11.01 piglets. When the number of piglets born was compared between surrogate sows and fertilized sows, the results indicated that the farrow rate in surrogate sows was significantly lower (*P*<0.0001 in t tests). However, even for fertilized eggs, embryonic mortality in the domestic pig is naturally quite high and approximately 30 to 50% of the ova released from the ovary are fail to survive during gestation (Zavy and Geisert, 1994). Considering the common phenomenon of polyspermic fertilization in pig (Suzuki et al., 2003), the result possibly further illustrates the importance for the quality of transferred embryos.

When a Life Table Survival Analysis was applied, the median survival was observed to correspond to Day 117.5 and Day 115 in surrogate and normally fertilized sows, respectively (Fig. 2D). On Day 125 and Day 117, the proportions of survival in surrogate and normally fertilized sows reached 95%, indicating that the gestation was accomplished from a statistical point of view and Caesarean delivery should be performed after these time point because there might be stillbirth. Additionally, a previous report showed that the piglet mortality rate following vaginal delivery was higher than that following Caesarean section (Schmidt et al., 2011). Therefore, it appears that Caesarean operation for surrogate sows should preferentially be performed earlier than Day 125. However, it should be noted here that there were also healthy piglets born after Day 125. Therefore, the causality between abnormality and even mortality in piglets and pregnancy length needs to be further examined.

In addition, the length of pregnancy for surrogate sows and their average litter size shows a negative correlation (supplementary material Table S12). As revealed in supplementary material Table S10, it could be found that most of surrogate sows delivered piglets between Day 114 to Day 123. To better understand the occurrence of cloned piglets delivery, this interval could be further divided into two test groups if using the median survival point Day 118 as the divide. Thus, there were three stages, including early delivery (prior to Day 119) with a survival proportion lower than 60%, intermediate delivery (Day 119 to Day 123) with a survival proportion range from 60% to 90%, and late delivery (later than Day 123) with a survival proportion higher than 90%; the average litter size in the early delivery group can be observed to be higher than that in both the middle and late delivery groups (supplementary material Table S11).

### Other potential factors

Due to the purpose of the embryo transfer was to make transgenic animals, there is possibility that the different genetic modifications would affect the cloning efficiency. Actually, a recent report showed that there was no significant difference regarding to cloning efficiency, pregnancy and delivery rate among three classes of gene modifications: additive gene transfer, homologous recombination and replication of already existing transgenic pigs (Kurome et al., 2013). In the present study, 93% (431/462) embryo transfers belong to “additive gene transfer” and 7% (31/462) embryo transfers belong to “homologous recombination”; there is also no significant difference in PR rate between them. Additionally, the goal of our laboratory is to optimize the porcine reproductive performance and the selected transgenes were with no apparent cytotoxicity, such as Cre-inducible EGFP (Li et al., 2009), Cre recombinase (Chen et al., 2010) and human apolipoprotein CIII (Wei et al., 2012). Therefore, it is believed that the different genetic modifications would not largely affect the statistical analysis in this study. The process of somatic cell nuclear transfer can be affected by multiple factors and there are also some other potential factors which were not being evaluated here, such as different type of donor cells (only fetal fibroblast cells in the present study) and even different team members for manipulation. Whatever, we believe that our conclusions would serve the craft of producing cloned pigs.

## Supplementary Material

Supplementary Material

## References

[b1] Chen L. Li L. Pang D. Li Z. Wang T. Zhang M. Song N. Yan S. Lai L. X. Ouyang H. (2010). Construction of transgenic swine with induced expression of Cre recombinase. Animal 4, 767–771 10.1017/S1751731109991571 22444131

[b2] Evans J. J. Anderson G. M. (2012). Balancing ovulation and anovulation: integration of the reproductive and energy balance axes by neuropeptides. Hum. Reprod. Update 18, 313–332 10.1093/humupd/dms004 22442260

[b3] Gonzalez-Bulnes A. Astiz S. Encinas T. Gonzalez-Añover P. Perez-Solana M. Sanchez-Sanchez R. Torres-Rovira L. Tresguerres J. A. (2012). Characterization of a distinctive pattern of periovulatory leptin secretion and its relationship with ovulation rate and luteal function in swine with obesity/leptin resistance. Peptides 37, 290–293 10.1016/j.peptides.2012.07.016 22841857

[b4] Hoshino Y. Uchida M. Shimatsu Y. Miyake M. Nagao Y. Minami N. Yamada M. Imai H. (2005). Developmental competence of somatic cell nuclear transfer embryos reconstructed from oocytes matured *in vitro* with follicle shells in miniature pig. Cloning Stem Cells 7, 17–26 10.1089/clo.2005.7.17 15996114

[b5] Hurtgen J. P. Leman A. D. (1980). Seasonal influence on the fertility of sows and gilts. J. Am. Vet. Med. Assoc. 177, 631–635. 7440358

[b6] Koo O. J. Kang J. T. Kwon D. K. Park H. J. Lee B. C. (2010). Influence of ovulation status, seasonality and embryo transfer method on development of cloned porcine embryos. Reprod. Domest. Anim. 45, 773–778 10.1111/j.1439-0531.2009.01346.x 19281594

[b7] Kun Z. Shaohua W. Yufang M. Yankun L. Hengxi W. Xiuzhu S. Yonghui Z. Yan L. Yunping D. Lei Z. (2007). Effects of leptin supplementation in *in vitro* maturation medium on meiotic maturation of oocytes and preimplantation development of parthenogenetic and cloned embryos in pigs. Anim. Reprod. Sci. 101, 85–96 10.1016/j.anireprosci.2006.08.021 17161925

[b8] Kurome M. Geistlinger L. Kessler B. Zakhartchenko V. Klymiuk N. Wuensch A. Richter A. Baehr A. Kraehe K. Burkhardt K. (2013). Factors influencing the efficiency of generating genetically engineered pigs by nuclear transfer: multi-factorial analysis of a large data set. BMC Biotechnol. 13, 43 10.1186/1472-6750-13-43 23688045 PMC3691671

[b9] Lai L. X. Prather R. S. (2003). Production of cloned pigs by using somatic cells as donors. Cloning Stem Cells 5, 233–241 10.1089/153623003772032754 14733743

[b10] Lai L. Kolber-Simonds D. Park K. W. Cheong H. T. Greenstein J. L. Im G. S. Samuel M. Bonk A. Rieke A. Day B. N. (2002). Production of alpha-1,3-galactosyltransferase knockout pigs by nuclear transfer cloning. Science 295, 1089–1092 10.1126/science.1068228 11778012

[b11] Lai L. X. Kang J. X. Li R. F. Wang J. D. Witt W. T. Yong H. Y. Hao Y. H. Wax D. M. Murphy C. N. Rieke A. (2006). Generation of cloned transgenic pigs rich in omega-3 fatty acids. Nat. Biotechnol. 24, 435–436 10.1038/nbt1198 16565727 PMC2976610

[b12] Li R. Lai L. Wax D. Hao Y. Murphy C. N. Rieke A. Samuel M. Linville M. L. Korte S. W. Evans R. W. (2006). Cloned transgenic swine via *in vitro* production and cryopreservation. Biol. Reprod. 75, 226–230 10.1095/biolreprod.106.052514 16672718

[b13] Li L. Pang D. Wang T. Li Z. Chen L. Zhang M. Song N. Nie D. Chen Z. Lai L. (2009). Production of a reporter transgenic pig for monitoring Cre recombinase activity. Biochem. Biophys. Res. Commun. 382, 232–235 10.1016/j.bbrc.2009.02.146 19268654

[b14] Love R. J. Evans G. Klupiec C. (1993). Seasonal effects on fertility in gilts and sows. J. Reprod. Fertil. Suppl. 48, 191–206. 8145204

[b15] Ma Y. Li Y. Wei H. Li Q. Fang R. Zhao R. Zhang K. Xue K. Lou Y. Dai Y. (2009). Effects of chemical activation and season on birth efficiency of cloned pigs. Sci. China C Life Sci. 52, 657–664 10.1007/s11427-009-0087-3 19641871

[b16] Park K. W. Cheong H. T. Lai L. X. Im G. S. Kühholzer B. Bonk A. Samuel M. Rieke A. Day B. N. Murphy C. N. (2001). Production of nuclear transfer-derived swine that express the enhanced green fluorescent protein. Anim. Biotechnol. 12, 173–181 10.1081/ABIO-100108344 11808633

[b17] Polejaeva I. A. Chen S. H. Vaught T. D. Page R. L. Mullins J. Ball S. Dai Y. F. Boone J. Walker S. Ayares D. L. (2000). Cloned pigs produced by nuclear transfer from adult somatic cells. Nature 407, 86–90 10.1038/35024082 10993078

[b18] Polge C. Rowson L. E. Chang M. C. (1966). The effect of reducing the number of embryos during early stages of gestation on the maintenance of pregnancy in the pig. J. Reprod. Fertil. 12, 395–397 10.1530/jrf.0.0120395 5951131

[b19] Pope W. F. Lawyer M. S. Nara B. S. First N. L. (1986). Effect of asynchronous superinduction on embryo survival and range of blastocyst development in swine. Biol. Reprod. 35, 133–137 10.1095/biolreprod35.1.133 3741946

[b20] Rogers C. S. Stoltz D. A. Meyerholz D. K. Ostedgaard L. S. Rokhlina T. Taft P. J. Rogan M. P. Pezzulo A. A. Karp P. H. Itani O. A. (2008). Disruption of the CFTR gene produces a model of cystic fibrosis in newborn pigs. Science 321, 1837–1841 10.1126/science.1163600 18818360 PMC2570747

[b21] Schmidt M. Winter K. D. Dantzer V. Li J. Kragh P. M. Du Y. Lin L. Liu Y. Vajta G. Sangild P. T. (2011). Maternal endometrial oedema may increase perinatal mortality of cloned and transgenic piglets. Reprod. Fertil. Dev. 23, 645–653 10.1071/RD10220 21635813

[b22] Suzuki H. Saito Y. Kagawa N. Yang X. Z. (2003). *In vitro* fertilization and polyspermy in the pig: factors affecting fertilization rates and cytoskeletal reorganization of the oocyte. Microsc. Res. Tech. 61, 327–334 10.1002/jemt.10345 12811737

[b23] Suzuki S. Iwamoto M. Saito Y. Fuchimoto D. Sembon S. Suzuki M. Mikawa S. Hashimoto M. Aoki Y. Najima Y. (2012). Il2rg gene-targeted severe combined immunodeficiency pigs. Cell Stem Cell 10, 753–758 10.1016/j.stem.2012.04.021 22704516

[b24] Vajta G. Zhang Y. Macháty Z. (2007). Somatic cell nuclear transfer in pigs: recent achievements and future possibilities. Reprod. Fertil. Dev. 19, 403–423 10.1071/RD06089 17257528

[b25] Wei H. X. Zhang K. Ma Y. F. Li Y. Li Q. Y. Dai Y. P. Li N. (2009). Stage-dependent effect of leptin on development of porcine embryos derived from parthenogenetic activation and transgenic somatic cell nuclear transfer. J. Reprod. Dev. 55, 99–104 10.1262/jrd.19167 19008650

[b26] Wei J. Ouyang H. Wang Y. Pang D. Cong N. X. Wang T. Leng B. Li D. Li X. Wu R. (2012). Characterization of a hypertriglyceridemic transgenic miniature pig model expressing human apolipoprotein CIII. FEBS J. 279, 91–99 10.1111/j.1742-4658.2011.08401.x 22023023

[b27] Zavy M. T. Geisert R. D. (1994). Embryonic Mortality in Domestic Species Boca Raton, FL: CRC Press.

