# Supplementary material for: Efficiency of porcine somatic cell nuclear transfer – a retrospective study of factors related to embryo recipient and embryos transferred
Source: Biol Open. 2013 Oct 2;2(11):1223–8. doi: 10.1242/bio.20135983 (PMC3828769; doi:10.1242/bio.20135983)
Supplement: Supplementary Material [file supp_2_11_1223__index.html]

Efficiency of porcine somatic cell nuclear transfer – a retrospective study of factors related to embryo recipient and embryos transferred — Efficiency of porcine somatic cell nuclear transfer – a retrospective study of factors related to embryo recipient and embryos transferred — Efficiency of porcine somatic cell nuclear transfer – a retrospective study of factors related to embryo recipient and embryos transferred — Supplementary Material 

# Efficiency of porcine somatic cell nuclear transfer – a retrospective study of factors related to embryo recipient and embryos transferred

## bio.20135983 Supplementary Material

**Files in this Data Supplement:**

- Supplementary Material - Yongye Huang et al. doi: 10.1242/bio.20135983
