# Supplementary material for: Efficiency of porcine somatic cell nuclear transfer – a retrospective study of factors related to embryo recipient and embryos transferred
Source: Biol Open. 2013 Oct 2;2(11):1223–8. doi: 10.1242/bio.20135983 (PMC3828769; doi:10.1242/bio.20135983)
Supplement: Supplementary Material [file supp_bio.20135983_bio.20135983-s1.pdf]

Biology Open

Supplementary Material

Yongye Huang et al. doi: 10.1242/bio.20135983

Table S1. Statistics on full-term pregnancy in different years#.

Year	Surrogates	FTP (%)
2008	43	11 (25.6)
2009	111	24 (21.6)
2010	178	54 (30.3)
2011	105	20 (19.1)
2012	25	3 (12.0)
total	462	112 (24.2)

#The comparison of FTP rate among these years was made by Chi-square Test for R×C tables and the result was: $\chi^2=7.640$, $v=4$, $P=0.106$.

Table S2. Statistics on pregnancy in different months.

Month	Surrogates	PR (%)	*P* value#	FTP (%)	*P* value#
March	13	3 (23.1)	0.101	2 (15.4)	0.356
April	60	24 (40.0)	0.292	14 (23.3)	0.505
May	78	43 (55.1)	0.036	30 (38.5)	0.004
June	74	33 (44.6)	0.529	20 (27.0)	0.329
July	31	10 (32.3)	0.119	4 (12.9)	0.098
August	44	21 (47.7)	0.382	10 (22.7)	0.489
September	55	31 (56.4)	0.049	15 (27.3)	0.348
October	61	26 (42.6)	0.444	13 (21.3)	0.359
November	46	14 (30.4)	0.038	4 (8.7)	0.007
Total	462	205 (44.4)	0.051&	112 (24.2)	0.019&

#The *P* value was calculated by Binomial test, that is the number in each month compared with the average percentage, respectively.
&The *P* value was calculated by Chi-square test among the tested months.

Table S3. Comparison of FTP rate between May and other months by Chi-square tests#.

Month	χ^2 value	Variance	Sig. (2-sided)	Sig. (1-sided)
March	2.603	1	0.129	0.093
April	3.574	1	0.067	0.043*
June	2.249	1	0.167	0.092
July	6.752	1	0.011	0.007**
August	3.160	1	0.108	0.056
September	1.804	1	0.197	0.123
October	4.712	1	0.041	0.023*
November	12.882	1	0.000	0.000***

#The results were taken from continuity correction, which was computed only for 2×2 table.
*denoted significantly different.

Table S4. The designated initial date of each season from 2008 to 2012.

Year	Spring	Summer	Autumn	Winter
2008	2008.04.12	2008.06.11	2008.08.22	2008.10.23
2009	2009.04.26	2009.07.26	2009.08.20	2009.10.05
2010	2010.04.30	2010.06.02	2010.08.05	2010.10.10
2011	2011.05.08	2011.06.28	2011.08.15	2011.10.23
2012	2012.04.17	2012.06.24	–	–
Designated temperature*	from 10 to 22℃	above 22℃	from 22 to 10℃	below 10℃

*The Climate-Temperature Law was raised by Chinese scholar Baokun Zhang in 1934. For example, when five consecutive days and most of the following days had an average temperature above 22℃, the first day of the climate is designated as the initial day of summer.
– indicated that the date for that season was not designated.

Table S5. Statistics on pregnancy in winter.

Season	Surrogates	PR (%)	FTP (%)
1st Winter	78	28 (35.9)	11 (14.1)
2nd Winter	67	25 (37.3)	16 (23.9)
P value[&]	–	0.865	0.141

[&]The *P* value was calculated by Chi-square.

Table S6. Coefficients of linear regression among total, PR and FTP surrogate number in different months.

Model		Unstandardized coefficients		Standardized coefficients		
		B	Std. Error	Beta	t	Sig.
Total to FTP	constant	−7.387	4.055		−1.821	0.111
	Total	0.386	0.074	0.892	5.226	0.001
PR to FTP	constant	−3.326	1.828		−1.820	0.112
	PR	0.692	0.071	0.965	9.696	0.000
Total to PR	constant	−6.461	4.127		−1.566	0.161
	Total	0.570	0.075	0.944	7.572	0.000

Table S7. Statistics on embryos lost in different months.

Month	PR	FTP	BLO (%)	*P* value[#]
March	3	2	1 (33.3)	0.569
April	24	14	10 (41.7)	0.439
May	43	30	13 (30.2)	0.031
June	33	20	13 (39.4)	0.305
July	10	4	6 (60.0)	0.269
August	21	10	11 (52.4)	0.333
September	31	15	16 (51.6)	0.301
October	26	13	13 (50.0)	0.389
November	14	4	10 (71.4)	0.045
Total	205	112	93 (45.4)	0.226[&]

[#]The *P* value was calculated by Binomial test, that is the number in each month compared with the average percentage, respectively.
[&]The *P* value was calculated by Chi-square test among the tested months.

Table S8. The average number of transferred embryos from 2009 to 2012.

Year	Total average	FTP	Non-FTP	Litter size
2009	241±40	249±40[a]	239±40[a]	2.04
2010	248±50	237±22[a]	252±56[a]	3.00
2011	238±48	267±54[a]	230±42[a]	3.37
2012	262±66	261±74[a]	263±66[a]	3.00

[a]Denotes that there were no significant differences existing within the same group.

Table S9. The abortion numbers of each day point after embryo transfer.

Day	Abortion number	Survival proportions (%)
Day 19	1	97.87
Day 20	2	93.62
Day 21	1	91.49
Day 23	2	87.23
Day 25	1	85.11
Day 26	1	82.98
Day 27	3	76.60
Day 28	5	65.96
Day 30	4	57.45
Day 31	3	51.06
Day 32	5	40.43
Day 33	6	27.66
Day 34	5	17.02
Day 35	1	14.89
Day 36	2	10.64
Day 38	1	8.51
Day 45	2	4.26
Day 67	2	0.00

Table S10. Statistics on the length of pregnancy for cloned pigs*.

	Cloned pigs delivery from 2008 to 2012			Normal pigs delivery in 2011		
Length of pregnancy	Number of surrogate sow	Total born piglets	Average born piglets	Number of sow	Total born piglets	Average born piglets
110	0	0	0.00	1	9	9.00
111	0	0	0.00	3	32	10.67
112	0	0	0.00	5	59	11.80
113	1	3	3.00	23	227	9.87
114	4	16	4.00	29	327	11.28
115	8	30	3.75	43	517	12.02
116	15	49	3.27	28	285	10.18
117	15	54	3.60	15	156	10.40
118	9	34	3.78	4	53	13.25
119	10	28	2.80	1	3	3.00
120	3	11	3.67	0	0	0.00
121	7	13	1.86	1	18	18.00
122	4	10	2.50	1	9	9.00
123	4	10	2.50	0	0	0.00
124	1	1	1.00	0	0	0.00
125	1	4	4.00	0	0	0.00
127	1	1	1.00	0	0	0.00
129	2	2	1.00	0	0	0.00
132	1	1	1.00	0	0	0.00
Total	86	267	3.10	154	1695	11.01

*The piglets born by caesarean delivery are not included.

Table S11. Statistics for the different length stages of pregnancy for cloned pigs.

Group	Early	Middle	Late
Total born piglets	186	72	9
Surrogate sow number	52^{a}	28^{b}	6^{c}
Average litter size	3.58^{a}	2.57^{b}	1.5^{b}

^{a,b,c}Values with different superscripts are significantly different within the same group. The corresponding number in each day point of different stage was compared by One-way ANOVA.

Table S12. Results of bivariate correlations analysis.

	Pearson		Spearman	
Group	Coefficient	Significance	Coefficient	Significance
Surrogates and PR in months	0.944	0.000	0.933	0.000
Surrogates and FTP in months	0.900	0.000	0.900	0.000
PR and FTP in months	0.965	0.000	0.979	0.000
Length of pregnancy and born cloned piglets per litter	−0.578	0.000	−0.381	0.010
Length of pregnancy and born fertilized piglets per litter	−0.139	0.650	−0.099	0.748
Transferred embryos number and surrogate sow litter size	−0.012	0.917	0.054	0.647
